# Lactate dehydrogenase and hemolysis index to predict vaso-occlusive crisis in sickle cell disease

**DOI:** 10.1038/s41598-023-48324-w

**Published:** 2023-12-01

**Authors:** Guillaume Feugray, Cécile Dumesnil, Maximilien Grall, Ygal Benhamou, Helene Girot, Julie Fettig, Valery Brunel, Paul Billoir

**Affiliations:** 1 Department of General Biochemistry, UNIROUEN, INSERM U1096, CHU Rouen, Normandie Univ, 76000 Rouen, France; 2 Department of Pediatric Onco-Hematology, CHU Rouen, 76000 Rouen, France; 3 Department of Internal Medicine, CHU Rouen, 76000 Rouen, France; 4 Department of Internal Medicine, UNIROUEN, INSERM U1096, CHU Rouen, Normandie Univ, 76000 Rouen, France; 5 Department of General Biochemistry, CHU Rouen, 76000 Rouen, France; 6 UNIROUEN, INSERM U1096, CHU Rouen, Vascular Hemostasis Unit, Normandie Univ, 76000 Rouen, France; 7 Service de Biochimie Générale, Centre Hospitalier Universitaire Charles Nicolle, 1 Rue de Germont, 76031 Rouen, France

**Keywords:** Biochemistry, Biomarkers, Risk factors

## Abstract

Sickle cell disease (SCD) is an inherited hemoglobinopathy disorder associated with chronic hemolysis. A major complication is vaso-occlusive crisis (VOC), associating frequent hospitalization, morbidity and mortality. The aim of this study was to investigate whether hemolysis biomarkers were able to predict VOC risk in adult patients with SCD requiring hospitalization within 1 year. This single-center prospective study included adult patients with SCD at steady state or during VOC. A total of 182 patients with SCD were included, 151 at steady state and 31 during VOC. Among the 151 patients at steady state 41 experienced VOC within 1 year (median: 3.0 months [2.0–6.5]). We observed an increase of lactate dehydrogenase (LDH) (*p* = 0.01) and hemolysis index (HI) (*p* = 0.0043) during VOC compared to steady state. Regarding patients with VOC requiring hospitalization, LDH (*p* = 0.0073) and HI (*p* = 0.04) were increased. In unadjusted logistic regression, LDH > median (> 260 U/L) (RR = 3.6 [1.29–10.88], *p* = 0.0098) and HI > median (> 8 UA/L) (RR = 3.13 [1.91–5.33]; *p* < 0.001) were associated with VOC. The association of LDH > 260 U/L and HI > 12 UA/L presented a sensitivity of 90%, and a specificity of 72.9% to predict VOC. The association of LDH and HI cut-off was able to predict VOC risk in SCD.

## Introduction

Sickle cell disease (SCD) is the most common genetic disease worldwide. SCD is an inherited hemoglobinopathy disorder due to *HBB* gene mutation leading to amino-acid substitution on the β globin chain. The main outcome is synthesis of abnormal hemoglobin S (HbS) which polymerizes in the deoxygenated state and causes hemolytic anemia and painful vaso-occlusive crisis (VOC) leading to severe complications such as frequent hospitalization, acute chest syndrome (ACS), organ damage or death^1,2^.

Different hemolysis parameters, including lactate dehydrogenase (LDH), are frequently used in the medical care of SCD to determine the severity of hemolysis^3^. Several phenotypes have been characterized and are considered as hyper-hemolysis^3,4^. In recent years, a hemolysis index (HI) has been developed by several manufacturers to estimate hemolysis as a quality measure of samples to detect both pre-analytical and analytical interferences. Agreement between international unit and mg/dL for HI has been reported^5^. Several comparisons between manufacturers have also been carried out^6^. Recent studies have demonstrated the use of coagulation or erythrocytic parameters to predict VOC^7,8^.

The aim of this study was to investigate whether hemolysis biomarkers, including lactate dehydrogenase and hemolytic index, were able to predict VOC risk in adult patients with SCD requiring hospitalization within 1 year.

## Results

### Demographic characteristics

**Figure 1:**
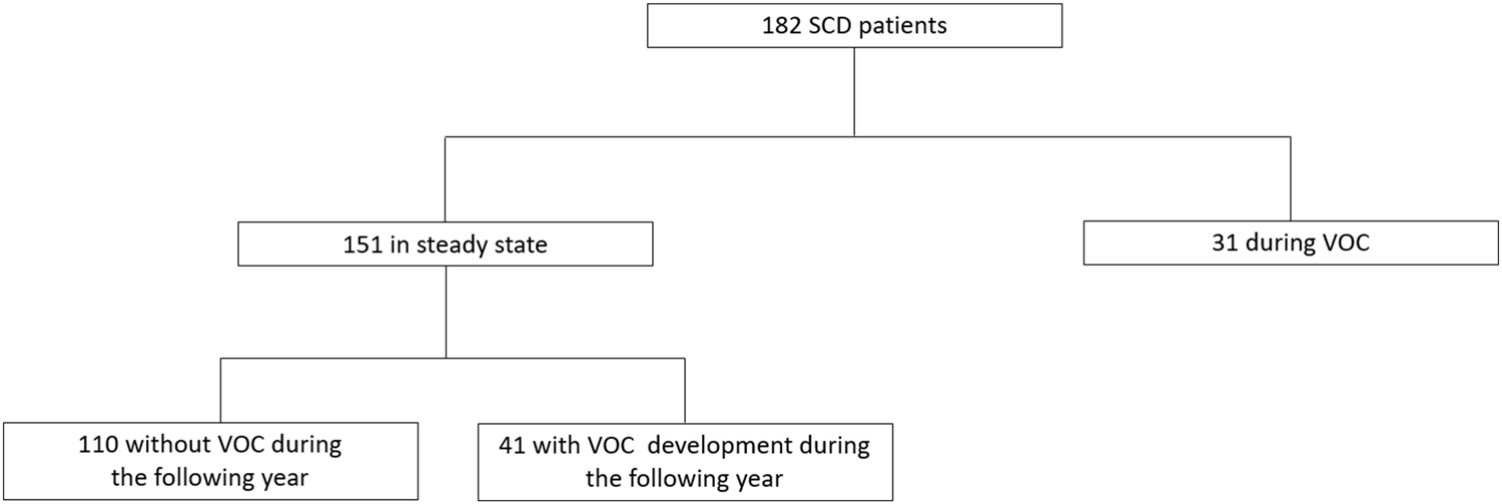
Flowchart of the study design.

A total of 182 patients with SCD were included in this study, 151 patients at steady state and 31 patients during VOC (Fig. 1). Among the 151 patients at steady state 62 (41.06%) were S/S–S/β^0^, 41 (27.15%) were S/Sα^3.7^ and 48 (31.78%) were S/C or S/β^+^. All genotypes were represented in VOC. Patients’ characteristics are summarized in Table 1. Among these 151 patients at steady state, 41 developed VOC within 1 year (median: 3.0 months [2.0–6.5]). In the VOC group, patients were younger (35.6 ± 11.8 vs. 31.3 ± 9.1 years, *p* = 0.041) and reported less osteonecrosis (33.7 vs. 12.9%, *p* = 0.03) and more splenectomy (3.3 vs. 16.1%, *p* = 0.01). In the VOC group, we observed a decrease of red blood cells (RBC) (*p* = 0.014) associated with an increase of mean corpuscular volume (MPC) (*p* = 0.036), leukocytes (*p* < 0.001), neutrophils (*p* < 0.001), monocytes (*p* = 0.036), reticulocytes (*p* = 0.008), LDH (*p* = 0.01), HI (*p* = 0.0043), C reactive protein (CRP) (*p* = 0.0015), proteinemia (*p* = 0.007), aspartate amino transferase (ASAT) (*p* = 0.022) and alanine amino transferase (ALAT) (*p* = 0.032).

**Table 1:** Characteristics of study population.

	SCD at steady state n = 151	SCD during VOC n = 31	*p* value
Clinical characteristics			
Age (years)	35.6 ± 11.8	31.3 ± 9.1	**0**.**041**
Male n (%)	70 (46.4)	16 (51.6)	0.69
Hydroxyurea n (%)	83 (54.9)	22 (70.9)	0.11
Osteonecrosis n (%)	51 (33.7)	4 (12.9)	**0**.**03**
Retinopathy n (%)	36 (23.8)	12 (38.7)	0.11
Vasculopathy n (%)	24 (15.9)	5 (16.1)	> 0.99
ACS n (%)	57 (37.8)	9 (29)	0.41
Cholecystectomy n (%)	69 (45.7)	9 (29.0)	0.11
Splenectomy n (%)	5 (3.3)	5 (16.1)	**0**.**01**
S/S–S/β^0^	62 (41.06)	18 (58.06)	0.11
S/Sα^3.7^	41 (27.15)	6 (19.35)	0.49
S/C – S/β^+^	48 (31.79)	7 (22.58)	0.39
Blood counts			
RBC (10^12^/L)	3.16 [2.70–4.09]	2.79 [2.44–3.59]	**0**.**014**
Hemoglobin (g/dL)	9.5 [8.1–10.6]	9.0 [8.0–10.2]	0.31
Hematocrit (%)	28.0 [23.7–32.0]	25.0 [23.0–29.0]	0.19
MCV (fL)	83.3 [75.1–92.4]	91.0 [79.9–99.3]	**0**.**036**
MCHC (g/dL)	34.5 [33.0–35.6]	34.9 [33.8–35.8]	0.12
Platelets (10^9^/L)	302 [187–399]	310 [248–399]	0.16
Leukocytes (10^9^/L)	7.3 [5.6–9.4]	10.5 [8.0–12.3]	**< 0**.**001**
Neutrophils (10^9^/L)	3.90 [2.72–5.15]	5.65 [4.11–7.46]	**< 0**.**001**
Lymphocytes (10^9^/L)	2.30 [1.54–3.04]	2.64 [1.98–3.55]	0.075
Monocytes (10^9^/L)	0.69 [0.48–1.02]	0.88 [0.55–1.31]	**0**.**036**
Hemolysis parameters			
Reticulocytes (10^9^/L)	188.8 [137.8–296.7]	288.1 [183.8–383.2]	**0**.**008**
Total bilirubin (µmol/L)	25.0 [17.5–44.0]	36.0 [18.5–50.0]	0.18
Indirect bilirubin (µmol/L)	24.5 [16.0–37.5]	29.0 [18.0–44.0]	0.42
LDH (U/L)	342 [243–448]	431 [364–502]	**0**.**01**
HI (UA/L)	8 [1–17]	12 [8–42]	**0**.**0043**
Biochemistry parameters			
CRP (mg/L)	5.0 [2.0–8.5]	14.0 [6.0–20.0]	**0**.**0015**
Urea (mmol/L)	3.1 [2.4–4.2]	3.3 [2.2–4.1]	0.72
Creatinine (µmol/L)	59 [50–72]	53 [41–81]	0.2
Proteinemia (g/L)	78.0 [74.5–81.0]	76.0 [70.0–77.0]	**0**.**007**
ALP (U/L)	72.5 [59.0–99.5]	85.0 [62.0–123.0]	0.33
ASAT (U/L)	35.5 [26.8–48.0]	43.0 [35.5–62.5]	**0**.**022**
ALAT (U/L)	22.0 [15.0–32.3]	31.0 [20.0–66.0]	**0**.**032**
yGT (U/L)	36.0 [24.0–73.0]	57.5 [22.5–109.5]	0.24

Data are expressed as median and [IQR] except for age (mean ± SD) and clinical characteristics with n is the total number of patients (%). ACS, acute chest syndrome; RBC, red blood cells; MCV, mean corpuscular volume; MCHC, mean corpuscular hemoglobin concentration; LDH, lactate dehydrogenase; HI, Hemolysis Index; CRP, C-reactive protein; ALP, alkaline phosphate; ASAT, aspartate amino transferase; ALAT, alanine amino transferase; yGT, gamma-glutamyl transferase.

Significant values are in [bold].

### Correlation

**Figure 2:**
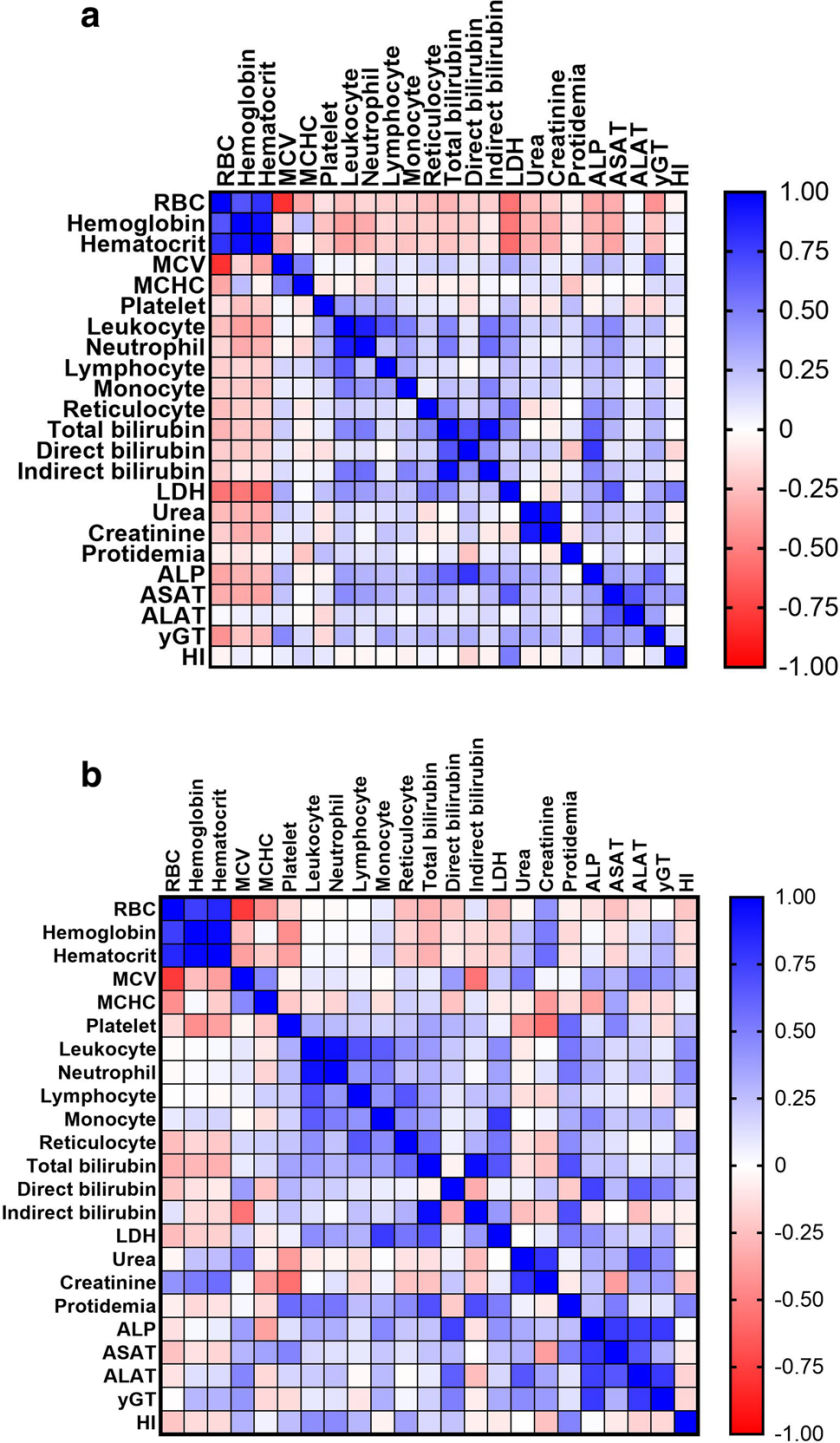
Correlation of hemolysis parameters in all SCD genotypes at steady state (**a**) and during vaso-occlusive crisis (**b**). Data are expressed as r Pearson correlation and (*p* value). RBC, red blood cells; MCV, mean corpuscular volume; MCHC, mean corpuscular hemoglobin concentration; LDH, lactate dehydrogenase; ALP, alkaline phosphate; ASAT, aspartate amino transferase; ALAT, alanine amino transferase; yGT, gamma-glutamyl transferase; HI, Hemolysis Index.

The correlation between all biomarkers was estimated, in particular hemolysis parameters, during steady state and VOC (Fig. 2).

At steady state, LDH was correlated with RBC (r = − 0.54, *p* < 0.0001), hemoglobin (r = − 0.53, *p* < 0.0001), hematocrit (r = − 0.57, *p* < 0.0001), mean corpuscular volume (MCV) (r = 0.33, *p* = 0.0003), platelets (r = 0.24, *p* = 0083), leukocytes (r = 0.43, *p* < 0.0001), neutrophils (r = 0.38, *p* < 0.0001), monocytes (r = 0.21, *p* = 0.02), reticulocytes (r = 0.49, *p* < 0.0001), total bilirubin (r = 0.44, *p* < 0.0001), indirect bilirubin (r = 0.25, *p* = 0.03), alkaline phosphatase (ALP) (r = 0.35, *p* = 0.0008), ASAT (r = 0.64, *p* < 0.0001) and HI (r = 0.51, *p* < 0.0001). HI was correlated with ASAT (r = 0.38, *p* < 0.0001).

At VOC, LDH was correlated with monocytes (r = 0.77, *p* < 0.0001), reticulocytes (r = 0.53, *p* = 0.02) and total bilirubin (r = 0.65, *p* = 0.0022). HI was correlated with leukocytes (r = 0.44, *p* = 0.015), and neutrophils (r = 0.45, *p* = 0.011).

### VOC prediction

Among the 151 patients included at steady state, 41 patients were hospitalized for VOC within 1 year. Results are reported in Table 2. In these patients, significant increases were observed in LDH (*p* = 0.0073), and HI (*p* = 0.04). We evaluated biomarkers to predict VOC. In unadjusted logistic regression, LDH > median (> 260 U/L) (RR = 3.6 [1.29–10.88], *p* = 0.0098) and HI > median (> 8 UA/L) (RR = 3.13 [1.91–5.33]; *p* < 0.001) were associated with VOC. To increase specificity, a Receiver Operating Characteristic (ROC) curve was built to determine VOC risk: HI > 12 UA/L (area under the curve (AUC): 0.61, sensitivity: 46.0%, specificity: 69.6%).

**Table 2:** Comparison of biologic parameters in SCD patients developing or not VOC within 1 year.

	SCD at steady state without VOC development n = 110	SCD at steady state with VOC development n = 41	*p* value
Hydroxyurea n (%)	51 (46.4)	32 (78)	**0**.**004**
Blood counts			
RBC (10^12^/L)	3.24 [2.82–4.25]	3.02 [2.52–3.73]	**0**.**027**
Hemoglobin (g/dL)	9.75 [8.40–10.68]	8.75 [7.90–10.08]	0.07
Hematocrit (%)	28 [24–32]	26 [23–30]	0.07
MCV (fL)	81.3 [72.9–90.8]	84.2 [80.9–95.1]	0.086
MCHC (g/dL)	34.7 [33.0–35.7]	34.3 [32.9–35.5]	0.69
Platelets (109/L)	290 [182–367]	340 [199–40+]	0.10
Leukocytes (10^9^/L)	7.1 [5.7–8.7]	7.6 [5.110.0]	0.18
Neutrophils (10^9^/L)	3.85 [2.71–4.98]	4.09 [3.11–6.28]	0.09
Lymphocytes (10^9^/L)	2.30 [1.53–3.03]	2.20 [1.62–3.17]	0.77
Monocytes (10^9^/L)	0.67 [0.46–0.97]	0.78 [0.51–1.04]	0.31
Hemolysis parameters			
Reticulocytes (10^9^/L)	161 [121–275]	284 [201–367]	**0**.**0004**
Total bilirubin (µmol/L)	25.0 [17.0–42.3]	25.0 [18.0–46.0]	0.62
Indirect bilirubin (µmol/L)	25.0 [15.75–38.25]	24.0 [18.75–37.50]	0.56
LDH (U/L)	323 [213–417]	410 [311–530]	**0**.**0073**
HI (UA/L)	6.5 [5.0–16.0]	11.0 [5.0–21.0]	**0**.**04**
Biochemistry parameters			
CRP (mg/L)	5.0 [3.0–7.5]	6.0 [2.3–10.8]	0.31
Ferritin (µg/L)	114 [48–274]	161 [83–298]	0.33
Urea (mmol/L)	3.15 [2.40–4.58]	2.85 [2.30–3.60]	0.11
Creatinin (µmol/L)	63.0 [53.0–79.8]	55.0 [46.8–63.3]	**0**.**0072**
Proteinemia (g/L)	78 [75–81]	77 [73–81]	0.30
ALP (U/L)	69.5 [58.0–100.3]	78.0 [64.8–95.0]	0.49
ASAT (U/L)	34 [26–48]	38 [31–48]	0.41
ALAT (U/L)	23 [15–34]	20 [14–28]	0.28
yGT (U/L)	36 [24–74]	31 [21–73]	0.71

Data are expressed as median and [IQR], n is the total number of patients. RBC, red blood cells; MCV, mean corpuscular volume; MCHC, mean corpuscular hemoglobin concentration; LDH, lactate dehydrogenase; HI, Hemolysis Index; CRP, C-reactive protein; ALP, alkaline phosphate; ASAT, aspartate amino transferase; ALAT, alanine amino transferase; yGT, gamma-glutamyl transferase; VOC, vaso-occlusive crisis.

Significant values are in [bold].

**Figure 3:**
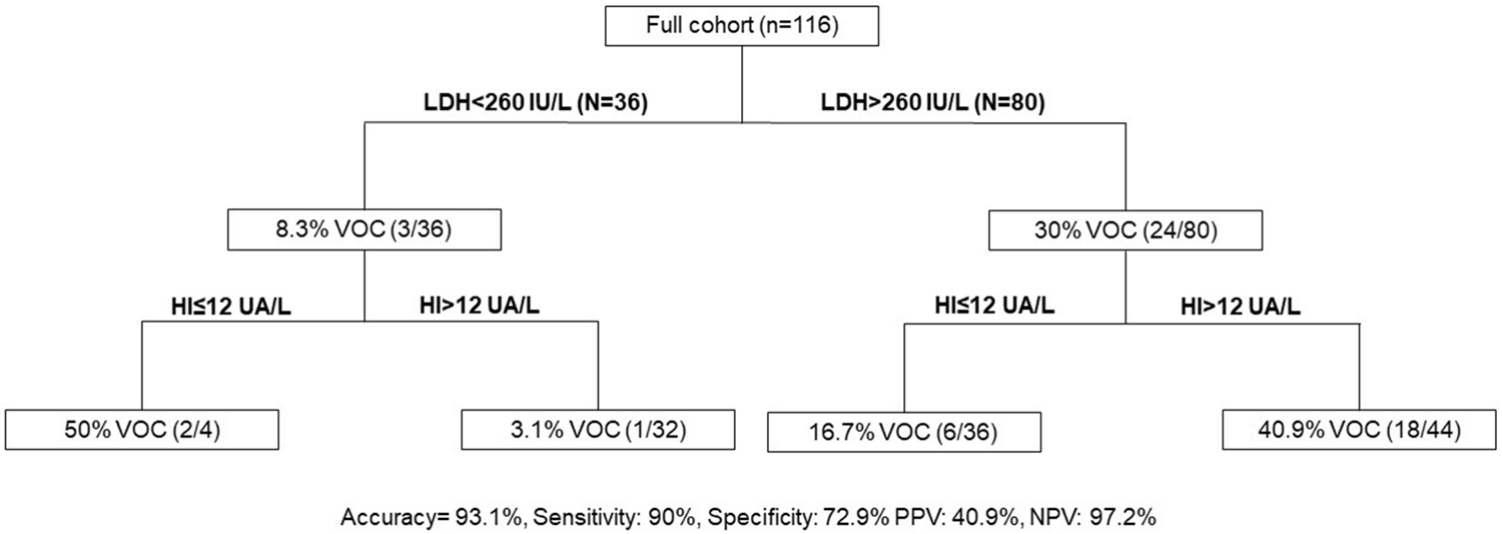
Algorithm to predict vaso-occlusive crisis. LDH, Lactate dehydrogenase; HI, Hemolysis Index; PPV, Positive Predictive Value. NPV, Negative Predictive Value. VOC, Vaso-occlusive crisis. Patients under classification tree is performed when both biomarkers is realized.

The association of LDH > 260 U/L and HI > 12 UA/L presented a sensitivity of 90.0% and a specificity of 72.9% to predict VOC development within 1 year in patients with SCD at steady state (Fig. 3).

## Discussion

The results of this study demonstrate a more severe hemolysis profile during VOC compared to steady state in SCD. Interestingly, we reported different correlations of LDH and HI during steady state and VOC. Moreover, patients with SCD who developed VOC requiring hospitalization within 1 year presented significantly higher LDH and HI. The association of these biomarkers, both easily available in routine laboratory practice, was able to predict VOC risk in SCD.

The underlying pathophysiology of SCD is complex, mainly due to chronic hemolysis. Nearly every organ can be affected. Clinical complications are multiple, notably sudden death, pulmonary hypertension, ischemic stroke^2^. It should be noted that SCD genotype presents phenotypic variability. Vaso-occlusive crisis is characterized by hemolytic anemia, endothelial damage, ischemic damage to tissues resulting in pain or organ failure with potentially life-threatening complications like ACS^2,9^. Thus, the search for biomarkers to predict severe complications is an active field of research^10,11^.

Recently, Saah et al*.* summarized the challenge to identify and validate prognostic biomarkers to assess an individual’s risk of specific clinical complications^12^. Here, we propose a specific biomarker to assess VOC risk. In SCD, hemolysis biomarkers at steady state can be useful to predict acute and/or chronic complications. LDH activity at steady state in patients with SCD is usually elevated, but the level depends on the type of SCD and on the type of disease-modifying treatments the patients are receiving^3^. As reported, long-term hydroxyurea treatment reduced LDH activity^13^. LDH significantly increases during acute painful VOC compared with steady state, whatever the severity of complications^14^. In our study, we demonstrated that VOC is associated with significant lower RBC and higher leukocytes, neutrophils, monocytes, reticulocytes, LDH, HI, CRP, ASAT, ALAT and proteinemia.

Moreover, during VOC, LDH activity is associated with severe complications like ACS requiring admission to intensive care unit, or leading to death^15^. Authors reported two cut-offs to predict severe outcome: LDH > 315 U/L (sensitivity of 100% and specificity of 27%) and LDH > 1000 U/L (sensitivity of 24% and specificity of 100%). However Alkindi et al. reported no association between ACS and LDH^16^. LDH activity is associated with delayed hemolytic transfusion reaction in patients transfused three weeks before VOC development^17^. Finally, LDH activity at steady state is correlated with pulmonary hypertension, priapism, leg ulceration and death^18^. However, the main limitation of LDH is that it is released from other organ injuries and is not specific to hemolysis with different iso-enzymes (LDH1-5)^19^.

Cell-free hemoglobin concentration, commonly referred to as HI, is an interesting tool to evaluate intravascular hemolysis which can predict clinical outcomes in hemolytic anemia^20^. The interest of HI in clinical care is extensive. Gils et al. reported that elevated HI was associated with a higher risk of cardiovascular disorders^21^. Mortality risk is significantly higher in patients with high HI than controls. Another interest of HI is for extracorporeal membrane oxygenation (ECMO) hemolysis monitoring^22,23^. An analysis demonstrated that HI > 50 mg/dL 24-h post ECMO was an independent predictor of mortality (OR = 3.4)^23^.Our results demonstrate significantly higher HI during VOC compared to steady state. Moreover, patients who developed VOC within 1 year reported higher HI. In unadjusted logistic regression, HI > 8 UA/L was associated with a RR of 3.13 to develop VOC. We also demonstrated a correlation between LDH, HI and other biomarkers. However, results show a difference between steady state and VOC.

The ability to predict VOC requiring hospitalization within 1 year in SCD could be an interesting tool in personalized medicine. Increased LDH and HI were associated with VOC development. Based on our results, LDH > 260 U/L and HI > 12 UA/L presented a sensitivity of 90.0%, a specificity of 72.9%, a PPV of 40.9% and a NPV of 97.2% to predict VOC development within 1 year. Previously, we demonstrated that thrombin generation assay and reticulocyte parameters allowed VOC prediction^7,8^. However, LDH and HI association reported higher sensitivity. The strength of this LDH and HI association is that it represents a simple, rapid, inexpensive and readily available tool in routine laboratory practice. Several scores to predict VOC severity in ACS are emerging^15,24^.

As a complement to hemolysis, heme and heme-related processing are strongly involved in the pathophysiology of SCD and represent promising biomarkers to investigate complications in SCD. Gentinetta et al. demonstrated that plasma-derived hemopexin inhibited heme-mediated cellular externalization of P-selectin and von Willebrand factor, and expression of IL-8, VCAM-1, and heme oxygenase-1 in cultured endothelial cells in a dose-responsive manner. Moreover, in a mouse model, intravenous injection of hemopexin prevented vaso-occlusion in a dose-dependent manner with good tolerance. These interesting results have led to a clinical trial of hemopexin in adults with SCD^25^. Associated with these results, Cardoso et al. reported that total extracellular heme levels, both plasma and serum, are correlated with the difference of hemoglobin between admission and convalescence and with SCD severity estimated by composite score^26^. Heme levels were neither associated with VOC severity or hemostasis activation. Moreover VOC is not characterized by significant increases in total extracellular heme levels^26^.

The main limitation of this work is the sample size. Indeed, these results are preliminary and need to be confirmed in a larger cohort with a subgroup analysis of genotypes. A longitudinal follow-up study in paired patients would enhance these results and refine the algorithm. Nevertheless, all genotypes are represented at steady state or during VOC and the algorithm can be used in both states. A second limitation is that we focused on VOC requiring hospitalization within 1 year. A prospective study is needed to determine an association between HI and other clinical complications like ACS, thromboembolism, osteonecrosis or retinopathy. Indeed, HI seems to be increasingly relevant both in terms of pre-analytical challenge and medical management. A third limitation is a lack of discrimination between in vivo and in vitro hemolysis, secondary to blood sample. However, currently, no assay is available to discriminate between the both.

## Conclusion

We propose a simple and inexpensive biomarker based on LDH and HI association to predict VOC risk in SCD. This biomarker is already available in routine laboratory practice. Hemolysis index represents an opportunity to study intravascular hemolysis in both pre-analytical conditions and in vivo.

## Materials and methods

### Study design

All patients included in the study were diagnosed and treated for SCD at Rouen University Hospital between September 2018 and April 2022. Patients at steady state were included during an annual visit. Patients in VOC were included less than 24 h after admission to emergency department.

All patients treated with hydroxyurea received therapy for at least 3 years. All patients received a systematic annual visit for medical care in which the number of VOC episodes was assessed.

Prospective data were collected and completed from medical records. The study was performed in accordance with the Declaration of Helsinki on biomedical research involving human subjects. The institutional review board (Rouen University Hospital) approved the study (Authorization protocol number: E2021-78) which is declared in clinical trials (clinical trials registration number: NCT05376046).

### Sample collection

Standard follow-up was performed using dipotassium EDTA tubes (BD Vacutainer EDTA, Plymouth) for blood counts. Plasma was obtained, 1700 g, 10 min at room temperature, from lithium heparin tubes (BD Vacutainer LH, Plymouth) for biochemistry parameters.

### Hemolysis index

The HI assay was carried out on a cobas 8000 chemistry analyzer (Roche Diagnostics, Mannheim, Germany) c701 or c502 module, using a spectrophotometric technique (SI2, Roche®). Hemolysis index is obtained using a formula calculated by the analyzer which takes into account: a two-color absorbance reading at 570 and 600 nm for hemolysis, a two-color absorbance reading at 660 and 700 nm for lipemia and correction factors. The measurement range is 5–1200 UA/L. The assay is fully automated with a 15 µL sample size.

### Standard hemolysis parameters

Samples were collected for routine follow-up. All biochemistry parameters were determined on a cobas 8000 chemistry analyzer (Roche Diagnostics, Mannheim, Germany). Hemolysis measurement was performed using plasma LDH, haptoglobin, total bilirubin (BT) and direct bilirubin (BD). Spectrophotometry was used for LDH measurement. Plasma samples with HI > 50 UA/L were excluded. Immunoturbidimetry was used for haptoglobin measurement. BT and BD were determined by colorimetric assay (Diazo method). Indirect bilirubin (BI) was calculated using the following formula: indirect bilirubin = total bilirubin − direct bilirubin.

### Other laboratory parameters

Blood counts were measured on XN-9000 (Sysmex, Villepinte, France). Hemoglobin phenotype was determined by high performance liquid chromatography (HPLC) (Variant II Biorad, California, United States), by capillary electrophoresis on Capillarys 3 Octa® (Kit hydragel hémoglobine, Sebia, Lisses, France) and iso-electrofocalization. Associated β thalassemia and C hemoglobin were considered as hemoglobin profile. The presence of α^3.7^ thalassemia was detected using a single-tube, multiplex-PCR assay.

### Statistical analysis

Statistical analyses were performed with GraphPad Prism for Windows, version 9.4.1. (GraphPad Software, San Diego, California, United States). Continuous variables were expressed as medians with interquartile range [IQR]. Mann–Whitney test, the Kruskal–Wallis test completed with two-stage step-up method of Benjamini, Krieger and Yekutieli and Fisher test were used. Univariate logistic regression analysis of VOC was performed using the following variables as predictors: LDH > median, HI > median. Receiver operating characteristic (ROC) curves were built for significant clinical characteristics. Pearson’s correlation was used to determine the correlation between two variables. *p* values < 0.05 were considered to be statistically significant.

### Informed consent

Informed consent was obtained from all individuals included in this study.

## Data Availability

All data generated or analyzed during this study are included in this published article.

## References

[1] Toledo SLDO, Guedes JVM, Alpoim PN, Rios DRA, Pinheiro MDB (2019). Sickle cell disease: Hemostatic and inflammatory changes, and their interrelation. Clin. Chim. Acta.

[2] Piel FB, Steinberg MH, Rees DC (2017). Sickle cell disease. N. Engl. J. Med..

[3] Stankovic Stojanovic K, Lionnet F (2016). Lactate dehydrogenase in sickle cell disease. Clin. Chim. Acta.

[4] Taylor JG (2008). Chronic hyper-hemolysis in sickle cell anemia: Association of vascular complications and mortality with less frequent vasoocclusive pain. PLoS ONE.

[5] Barbhuiya MA (2020). Automated measurement of plasma cell-free hemoglobin using the hemolysis index check function. J. Appl. Lab. Med..

[6] Lippi G (2009). Multicenter evaluation of the hemolysis index in automated clinical chemistry systems. Clin. Chem. Lab. Med..

[7] Feugray G (2022). Investigation of thrombin generation assay to predict vaso-occlusive crisis in adulthood with sickle cell disease. Front. Cardiovasc. Med..

[8] Feugray G (2022). Assessment of reticulocyte and erythrocyte parameters from automated blood counts in vaso-occlusive crisis on sickle cell disease. Front. Med..

[9] Rees DC, Williams TN, Gladwin MT (2010). Sickle-cell disease. Lancet.

[10] Njoku F (2023). Associations of hemolysis and anemia with cardiopulmonary dysfunction in an adult sickle cell disease cohort. Clin. Chim. Acta.

[11] Styles LA, Aarsman AJ, Vichinsky EP, Kuypers FA (2000). Secretory phospholipase A(2) predicts impending acute chest syndrome in sickle cell disease. Blood.

[12] Saah E, Fadaei P, Gurkan UA, Sheehan V (2022). Sickle cell disease pathophysiology and related molecular and biophysical biomarkers. Hematol. Oncol. Clin. North Am..

[13] Mecabo G, Yamamoto M, Biassi TP, Figueiredo MS (2015). Lactate dehydrogenase isoenzyme 3 and hemolysis in sickle cell anemia: A possible correlation?. Blood.

[14] Ballas SK, Marcolina MJ (2006). Hyperhemolysis during the evolution of uncomplicated acute painful episodes in patients with sickle cell anemia. Transfusion.

[15] Stankovic Stojanovic K (2012). High lactate dehydrogenase levels at admission for painful vaso-occlusive crisis is associated with severe outcome in adult SCD patients. Clin. Biochem..

[16] Alkindi S (2020). Predictors of impending acute chest syndrome in patients with sickle cell anaemia. Sci. Rep..

[17] Vidler JB, Gardner K, Amenyah K, Mijovic A, Thein SL (2015). Delayed haemolytic transfusion reaction in adults with sickle cell disease: A 5-year experience. Br. J. Haematol..

[18] Kato GJ (2006). Lactate dehydrogenase as a biomarker of hemolysis-associated nitric oxide resistance, priapism, leg ulceration, pulmonary hypertension, and death in patients with sickle cell disease. Blood.

[19] Ballas SK (2013). Lactate dehydrogenase and hemolysis in sickle cell disease. Blood.

[20] Lippi G, Favaloro EJ, Franchini M (2018). Haemolysis index for the screening of intravascular haemolysis: A novel diagnostic opportunity?. Blood Transfus..

[21] Gils C, Hansen DL, Nybo M, Frederiksen H (2023). Elevated hemolysis Index is associated with higher risk of cardiovascular diseases. Clin. Chem. Lab. Med..

[22] Bürki C, Volleberg M, Brunner D, Schmugge M, Hersberger M (2022). Using the hemolysis index of Abbott’s Alinity c for the measurement of plasma free hemoglobin in ECMO patients. Clin. Biochem..

[23] Omar HR (2015). Plasma free hemoglobin is an independent predictor of mortality among patients on extracorporeal membrane oxygenation support. PLoS ONE.

[24] Bartolucci P (2016). Score predicting acute chest syndrome during vaso-occlusive crises in adult sickle-cell disease patients. EBioMedicine.

[25] Gentinetta T (2022). Plasma-derived hemopexin as a candidate therapeutic agent for acute vaso-occlusion in sickle cell disease: Preclinical evidence. J. Clin. Med..

[26] Cardoso EC (2023). Changes in heme levels during acute vaso-occlusive crisis in sickle cell anemia. Hematol. Oncol. Stem Cell Ther..

